# miR-34a inhibits migration and invasion by regulating the SIRT1/p53 pathway in human SW480 cells

**DOI:** 10.3892/mmr.2015.3182

**Published:** 2015-01-12

**Authors:** MINGGUANG LAI, GANG DU, RUIYUE SHI, JUN YAO, GENHUA YANG, YUE WEI, DINGGUO ZHANG, ZHENGLEI XU, RU ZHANG, YINGXUE LI, ZICHENG LI, LISHENG WANG

**Affiliations:** 1 Department of Gastroenterology, Shenzhen People’s Hospital, Shenzhen, Guangdong 518020, P.R. China; 2 Department of Internal Medicine, First Affiliated Hospital of Jinan University, Guangzhou, Guangdong 510630, P.R. China

**Keywords:** microRNA-34a, SIRT1, p53, SW480 cells

## Abstract

MicroRNA-34a (miR-34a) is a direct transcriptional target of p53, and is downregulated in several different types of cancer. However, the underlying mechanism of the miR-34a effects in colorectal cancer is not well understood. In this study, we explored the role of miR-34a in cell invasion, migration, and apoptosis. Transient overexpression of miR-34a in SW480 cells caused a severe decrease in cell migration and invasion (both, p<0.05) compared to the control groups. Combining miR-34a transfection with 5-fluorouracil (5-FU) treatment further enhanced the inhibition in SW480 cell migration and invasion (both, p<0.05) compared to 5-FU treatment alone. These cellular changes were associated with upregulation of acetylated-p53 (ac-p53) and p21 and downregulation of sirtuin 1 (SIRT1). These data demonstrate that miR-34a regulates the expression of a number of critical proteins involved in apoptosis, proliferation and the response to chemotherapy. In summary, miR-34a increases the sensitivity of colon cancer cells to 5-FU treatment through specific regulation of the SIRT1/p53 pathway.

## Introduction

Colorectal cancer is one of the most prevalent types of cancer, with a high incidence of disease-related mortality and morbidity (1). The development of colorectal cancer is a multi-step process that is regulated by complex molecular networks. These networks are altered via sequential alterations in oncogenes, tumor-suppressor genes and non-coding RNAs (ncRNAs). microRNAs (miRs) are a type of ncRNA molecules, which negatively regulate protein expression at the post-transcriptional level by interacting with the 3′-untranslated region (3′-UTR) of target mRNAs and by inhibiting protein translation. Therefore, understanding the role of miRs is critical for defining cancer pathogenesis and developing new methods for diagnosis and treatment.

The family of miR-34 comprises some of the most studied miRs, which have been described as tumor suppressor genes in multiple cancer types including melanoma (2), pancreatic (3), prostate (4), colorectal (5) and non-small cell lung cancer (6) and neuroblastoma (7). miR-34a maps to the 1p36 genomic region in humans, and is expressed at higher levels compared to other family members. Several studies have indicated that upregulation of miR-34a can induce apoptosis, senescence, differentiation, cell-cycle arrest, and growth suppression (8–10). The abnormal expression of miR-34a results in cell-cycle arrest, growth inhibition and attenuated chemoresistance to antitumor drugs. It was previously suggested that miR-34a has a potential role in the treatment of p53-defective prostate cancer (4). miR-34a is also a promising therapeutic target for patients with hormone-refractory prostate cancer or patients showing drug resistance, where conventional chemical drug treatment exerts limited effects, or patients with distant tumor metastasis and recurrence (11). Sirtuin 1 (SIRT1) is a nicotinamide adenine dinucleotide (NAD)-dependent histone deacetylase, which has been associated with inflammation, circadian rhythm, hypoxic responses, cell survival, longevity and metabolic processes (12–15). SIRT1 is also involved in the mitochondrial antioxidant capacity, attenuating oxidative stress in coronary arterial endothelial cells (16). The tumor protein p53 is a sensor of chronic or acute alterations in cellular physiology, and more importantly, engages with DNA to maintain chromosomal integrity (17). The p53 levels are associated with those of miR-34a in keratinocytes (18), human mammary epithelial cells (19) and lymphoblast cell lines (20). miR-34a enhances p53 activity through a decrease in deacetylation, which in turn results in a decrease in SIRT1 expression. This decrease is achieved at the post-transcriptional level through binding to the 3′-UTR (21,22). In addition, inhibition of SIRT1 activates p53-dependent apoptosis via deacetylation and stabilization of p53. miR-34a-mediated inhibition of SIRT1 led to apoptosis in wild-type human colon cancer cells, while no apoptosis was observed in p53-deficient cancer cells (23). The positive feedback loop involving p53, SIRT1 and miR-34a may thus provide new therapeutic tools for the treatment of cancer.

However, the effect of the combination of miR-34a and chemotherapeutic drugs on colorectal cancer has rarely been syetematically explored. In addition, there are no reports investigating the synergistic effect of miR-34a with 5-fluorouracil (5-FU) on SW480 cells. In this study, we explored the effects of miR-34a in cell invasion, migration and apoptosis in SW480 cells. We further investigated the antitumor effect of both miR-34a and 5-FU in SW480 cells. Our experimental data provides evidence that miR-34a may be a suitable molecular target for colorectal cancer therapy. Finally, we examined the physiological pathway involving miR34a, p53 and SIRT1, which may be involved in the observed effects.

## Materials and methods

### Cell culture, transfection and treatments

SW480 cells were obtained from Nanfang Hospital, Southern Medical University (Guangzhou, China). The cells were cultured in Dulbecco’s modified Eagle’s medium (DMEM, HyClone Logan, UT, USA) with 10% fetal bovine serum (FBS; Sijiqing, Hangzhou, China) and 100 U/ml of penicillin and streptomycin, following standard procedures. Transfections were performed using Invitrogen™ Lipofectamine^®^ 2000 (Thermo Fisher Scientific, Waltham, MA, USA). Cells were treated as follows: negative control mimic (control group), 100 nM miR-34a mimic (miR-34a group), 200 μg/ml 5-FU (5-FU group), or 200 μg/ml 5-FU plus 100 nM miR-34a mimic (5-FU + miR-34a group) for 48 h. The negative control and the miR-34a mimics were obtained from GeneChem (Shanghai, China), and 5-FU was purchased from Jinyao Amino Acid Co., Ltd. (Tianjin, China).

### Reverse transcription-quantitative polymerase chain reaction (RT-qPCR)

Total RNA was extracted using TRIzol reagent (Thermo Fisher Scientific, Bremen, Germany) according to the manufacturer’s instructions. The first strand cDNA was synthesized by stem-loop primer reverse transcription reaction (Thermo Fisher Scientific). The following primer sequences were used: Hsa-miR-34a RT primer, 5′-CTCAACTGGTGTCGTGGAG TCGGCAATTCAGTTGAGACAACCAG-3′; sense: 5′-ACA CTCCAGCTGGGTGGCAGTGTCTTAG-3′; and antisense: 5′-CTCAACTGGTGTCGTGGAGTCG-3′ for Hsa-miR-34a; and sense: 5′-CTCGCTTCGGCAGCACA-3′ and antisense: 5′-AACGCTTCACGAATTTGCGT-3′ for U6. The real-time quantitative PCR kit (Fermentas, Helsingborg, Sweden) was used to facilitate the reactions. Reactions were conducted on an Applied BioSystems 7900HT thermocycler (Applied Biosystems, Inc.) and performed under the following thermal cycling conditions: 95°C for 10min, followed by 40 cycles of 95°C for 15 sec, 60°C for 30 sec and 72°C for 15 sec; followed by a 60°C for 1 min, 95°C for 15 sec. Raw data of all samples were normalized to that of the control and fold changes were calculated using a relative quantification equation (RQ=2^−ΔΔCt^).

### Western blot analysis

Western blot analysis was performed as previously described (24). Briefly, SW480 cells were homogenized in phosphate-buffered saline (PBS) containing a protease inhibitor cocktail (Beyotime Institute of Biotechnology, Shanghai, China). The samples were incubated overnight at 4°C with rabbit anti-p53 antibody, -acetyl p53, -SIRT1, or -acetyl p21 antibody (all from Cell Signaling Technology Inc., Danvers, MA, USA). The antibody signal was detected using a Chemiluminescent Detection kit according to the manufacturer’s protocol (Beyotime Institute of Biotechnology, Jiangsu, China). The relative band intensities in the blots were determined using the Adobe Photoshop software (Adobe Systems Inc., San Jose, CA, USA).

### Apoptosis analysis

Following treatment for 48 h as described above, SW480 cells were harvested, washed in ice-cold PBS, resuspended in 500 μl of binding buffer (C1062-2,Beyotime Institute of Biotechnology, Jiangsu, China) and incubated for 15 min in the dark with 5 μl of propidium iodide (PI; Beyotime Institute of Biotechnology, Jiangsu, China) and 5 μl of Annexin V-fluorescein isothiocyanate (FITC; Beyotime Institute of Biotechnology, Jiangsu, China). The samples were washed and resuspended in 500 μl PBS, and immediately analyzed by fluorescence-activated cell sorting (FACS) on a EPICS XL-MCL flow cytometer (Beckman Coulter, Brea, CA, USA).

### Cell cycle analysis

Following a 48 h treatment, SW480 cells were harvested, washed with PBS, and fixed in ice-cold 70% ethanol. Fixed cells were treated with DNase-free RNaseA (TransGen Biotech, Beijing, China) in PBS at 37°C for 30 min, followed by staining with PI at room temperature for 10 min. The proportion of cells at the different stages of the cell cycle was estimated by flow cytometry.

### Transwell cell migration assay

SW480 cells (48 h post-treatment) were trypsinized with 0.25% trypsin (Beyotime Institute of Biotechnology, Jiangsu, China) and suspended in serum-free DMEM at 5×10^5^ cells/ml. A total of 200 μl of the cell suspension were placed in the top chamber of a two-chamber Transwell assay system (Corning Inc., Corning, NY, USA) and 800 μl of medium containing 10% FBS were added in the lower chamber. Cells were cultured at 37°C for 12 h. The cells on the surface of the upper chamber were swapped and the cells under the surface of the lower chamber were stained with crystal violet (0.1%). Cell migration was evaluated by counting the cells that had migrated into the filters.

### Transwell cell invasion assay

Similar to the migration assay, 50 μl BD Matrigel™ (BD Biosciences, Franklin Lakes, NJ, USA) was added into each Transwell upper chamber and placed in a 37°C incubator for 2 h to solidify. The tumor cell invasive capacity was then assessed similarly to the cell migration assay.

### Statistical analysis

The results are expressed as mean ± standard deviation. Statistical significance was determined with Student’s t-tests (two-tailed, unpaired). P<0.05 was considered to indicate a statistically significant difference.

## Results

### miR-34a enhances the 5-FU effect on the SIRT1/p53 pathway in SW480 cells

**Figure 1:**
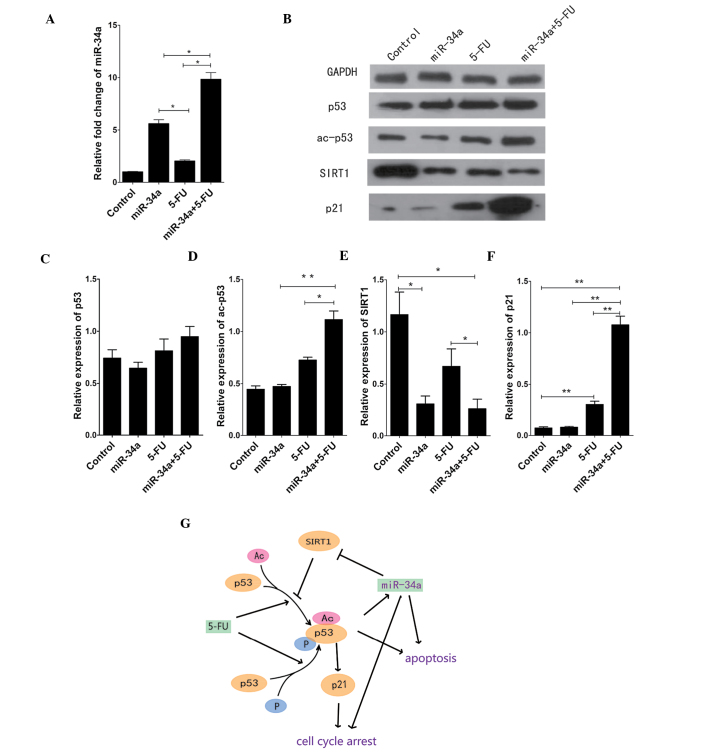
Activation of the miR-34a/SIRT1/p53 pathway by the miR-34a mimic and 5-FU. (A) Expression of miR-34a was determined by reverse transcription-quantitative PCR (RT-qPCR). (B) The different components of the miR-34a/SIRT1/p53 pathway were detected by western blotting with GAPDH as the loading control. (C–F) The relative expression of p53, ac-p53, SIRT1 and p21 was quantified from the western blot. (G) A model summarizing the findings from this study combined with previously published data, and describing the 5-FU/p53/p21 interactions. Quantitative data are presented as mean ± SD (n=3), with ^*^p<0.05 and ^**^p<0.01. miR, microRNA; ac, acetylated; 5-FU, 5-fluorouracil; SIRT1, sirtuin 1.

To first understand the effects of miR-34a, the expression level of this miR was measured by RT-qPCR in SW480 cells before and after transfection with the miR-34a mimic. The level of miR-34a in SW480 cells after transfection was markedly higher compared to the control group. In addition, the combination of the miR-34a mimic and 5-FU showed a synergistic effect on miR-34a expression (Fig. 1A). We also examined the protein expression of p53 and acetylated (ac)-p53 by western blot analysis. There was no significant change in the p53 level after treatment with the miR-34a mimic or 5-FU. The level of p53 was slightly but not significantly increased following combined treatment with the miR-34a mimic and 5-FU. By contrast, the combined treatment increased the level of ac-p53 compared to the control group (p<0.05), while no change was observed when miR-34a was used alone (Fig. 1B and D). To further understand the miR-34a pathway, we examined the protein expression of SIRT1 and p21, and found that SIRT1 expression is significantly decreased following treatment with miR-34a compared to the control group (p<0.05). 5-FU had a similar effect on SIRT1 expression in SW480 cells, with the changes being statistically significant (p<0.05) (Fig. 1B and E). In addition, the level of p21 was significantly and markedly changed by the combined miR-34a + 5-FU treatment compared to the other groups (p<0.01) (Fig. 1B and F). The results from western blot analysis were used in combination with published data to create a model illustrating the relationships among miR-34a, 5-FU and SIRT1/p53 (Fig. 1G).

### miR-34a induces apoptosis in SW480 cells and acts synergistically with 5-FU

**Figure 2:**
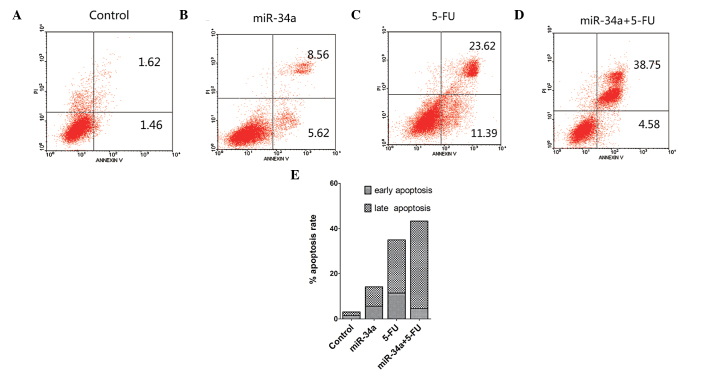
Effect of miR-34a and 5-FU on SW480 cell apoptosis. (A–D) Cells were stained with Annexin V-FITC/PI and analyzed by flow cytometry to determine the population of cells at the early and late apoptosis in the different treatment groups: negative control mimic-treated (control), miR-34a mimic-treated (miR-34a), 5-FU-treated (5-FU) and miR-34a mimic + 5-FU-treated (miR-34a + 5-FU). (E) The percentage of apoptotic cells in each group relative to the total number of cells was used to evaluate the apoptotic rates. miR, microRNA; 5-FU, 5-fluorouracil; FITC, fluorescein isothiocyanate; PI, propidium iodide.

To determine the effects of miR-34a and 5-FU treatment on cell death, we double-stained SW480 cells with Annexin V-FITC and PI and analyzed apoptosis by FACS at 48 h post-treatment. The shifts in cell population with the different treatments clearly indicated that the apoptotic rate of the miR-34a + 5-FU-treated group is higher than that of the miR-34a or the 5-FU group, while the control group showed the lowest rate of apoptosis (Fig. 2).

### miR-34a blocks the cell cycle in synergy with 5-FU

**Figure 3:**
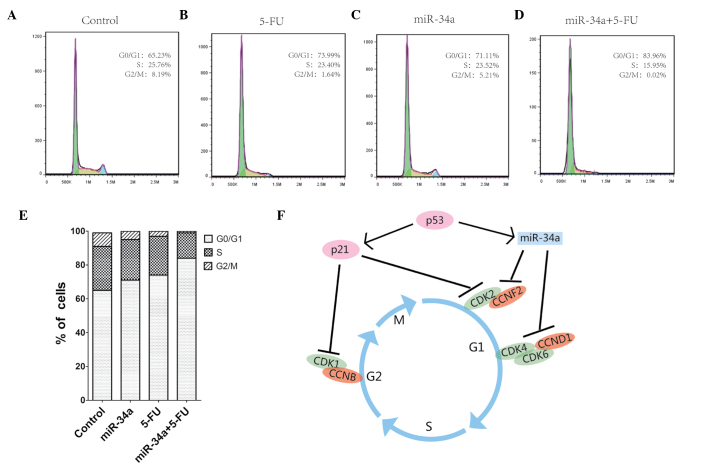
miR-34a inhibits the cycle phase transition from G0/G1 to S. (A–D) SW480 cells were treated with the negative control mimic (control), miR-34a mimic, 5-FU, or both, and were analyzed by flow cytometry to determine the percentage of cells in each of the different cell cycle phases, G1, S, and G2/M. (E) Bar diagram illutrating the distribution of cells from each group in the different cell cycle phases. (F) A model summarizing the findings from this study combined with previously published data, and describing the molecular interactions involved in cell cycle regulation. miR, microRNA; 5-FU, 5-fluorouracil; CDK, cyclin-dependent kinase; CCND1, cyclin D1.

We further examined the effect of miR-34a on the SW480 cell cycle by flow cutometry. This assay showed that both miR-34a and 5-FU block cell cycle progression and have a synergistic effect when combined. Individually, both miR-34a and 5-FU increased the percentage of cells detected at the G1 phase, and the combined treatment further increased this percentage, while the control group had the lowest proportion of G1-phase cells (Fig. 3A–D). The cell-cycle machinery involves the cyclin-dependent kinases (CDK)/cyclin complex, and p21 is known to suppress CDK1 activity via a p53-independent pathway. This event blocks cell progression into the G2/M phase. Moreover, p21 can block the progression of cells into the S phase, through inhibition of the CDK2 activity. miR-34a is involved in this cascade by blocking the progression of cells into the S phase through inhibition of the CDK2/4/6 activity (Fig. 3F).

### miR-34a inhibits migration and invasion of SW480 cells in vitro

**Figure 4:**
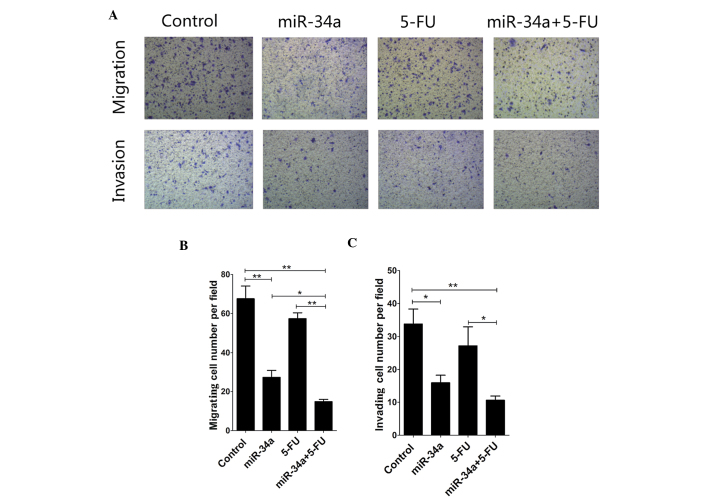
Migration and invasion of SW480 cells following different treatments. (A) Brightfield microscope images (magnification, ×20) of SW480 cells from the different treatment groups, and quantified data from the (B) migration and (C) invasion assays. Data are presented as mean ± SD (n=3), with ^*^p<0.05, ^**^p<0.01.

To investigate whether miR-34a also plays a role in cell migration and invasion, we tested if SW480 cells have the potential to digest Matrigel and migrate through the 8-mm membrane pores of a Transwell chamber. The Transwell tumor cell migration assay demonstrated that the miR-34a mimic-treated cells have a significantly reduced migrating capacity compared to the control group (p<0.01), and this effect was enhanced with the combined treatment miR-34a mimic + 5-FU (p<0.05) (Fig. 4A and B). The Transwell tumor cell invasion assay also showed that transfection with the miR-34a mimic significantly inhibits the invasive capacity of SW480 cells (p<0.05). This effect was enhanced with the miR-34a mimic + 5-FU combination, compared to 5-FU treatment alone (p<0.05) (Fig. 4A and C).

## Discussion

SW480 cells were used as a model to investigate the biological function of miR-34a in the context of colorectal cancer. The transient overexpression of miR-34a combined with 5-FU treatment reduced cell migration and invasion and increased apoptosis in these cells. These changes were most probably a result of the increased expression of ac-p53 and p21 and the decreased expression of SIRT1. These results demonstrate that miR-34a regulates the expression of critical proteins involved in cell apoptosis, proliferation and the response to chemotherapy. Moreover, miR-34a allowed sensitization of colon cancer cells to 5-FU, likely acting through the p53/SRIT1 pathway.

Typically, miRs are transcribed and processed in the nucleus to form pre-miRs. These pre-miRs are then exported to the cytoplasm and processed into miR duplexes. One strand from the duplex is incorporated into the miR-induced silencing complex (25). The study of miRs is a rapidly expanding research field, which includes investigation of their roles in tumor- or non-tumor-related diseases. miRs play an important role in cell function and fate in both the disease and the homeostatic states. These molecules are continuously reported as oncogenes or tumor suppressor genes. Not only are they detected in virtually every type of tumor, but they also display specific profiles in pathologies, which allow assessingmalignancy and evaluating the potential for metastasis (26). Increasing evidence has ascertained that a large number of miRs exhibit dysregulated expression in primary cancer specimens compared to tissues from healthy patient populations, including miR-21, miR-125b, miR-143, miR-145, miR-10b, miR-26a, miR-155 and miR-301 (27,28).

Although the tumor inhibition effect of miR-34a has been previously documented, its therapeutic potential on colorectal cancer remained unclear to date. Most research studies so far have focused on the role of miR-34a as a p53 transcriptional target and on its involvement in p53-mediated tumor suppression processes (29). Reduced or no miR-34a expression has been detected in a variety of tumors and cancer cell lines. To understand the role of miR-34a in SW480 cells, we tested the effects of miR-34a transfection on cell migration and invasion, and demonstrated that miR-34a inhibits SW480 cell migration and invasion; notably, this inhibition effect is enhanced when miR-34a transfection is combined with 5-FU treatment (Fig. 4). FACS analysis further showed indicated that miR-34a induces SW480 cell apoptosis,an effect again enhanced by 5-FU treatment (Fig. 2). These data overall suggest that miR-34a exerts an important antitumor effect on SW480 cells, which is similar to results reported in other studies on chronic lymphocytic leukemia (30), lung cancer (31), mesothelioma (10), neuroblastoma (32), prostate (33) and pancreatic cancer (34,35).

miR-34a induces cell cycle arrest by downregulating cell-cycle-related proteins such as cyclin D1 (CCND1), cyclin E2 (CCNE2), CDK4 and CDK6. Our data suggests that p53, p21 and miR-34a form a strong interaction network in the cell cycle (Fig. 3F). miR-34a has documented roles in the increase of acetylated p53 and in modification of p21 expression through the inhibition of SIRT1 expression. Our study confirmed that miR-34a significantly decreases the SIRT1 protein level, and the levels of ac-p53 and p21 were found particularly increased with the combined miR-34a + 5-FU treatment (Fig. 4). 5-FU is a pyrimidine antimetabolite cytotoxin, which induces DNA and RNA damage, resulting in cell death. 5-FU functions in a p53-dependent manner, likely causing changes in DNA metabolism and initiating events that culminate in the alteration of p53 expression (35). When the damaged cells cannot be repaired, p53 triggers cell elimination by inducing the expression of pro-apoptotic genes such as *Fas* and *Bax* (36). Interestingly, miR-34a can negatively regulate 5-FU resistance in human colorectal cancer DLD-1 cells by targeting the *SIRT1* and *E2F3* genes (37).

SIRT1 inactivates p53 by deacetylating a specific lysine residue to target it degradation. An increase in the miR-34a and a decrease in the SIRT1 levels were observed in leukemic cells that had been simultaneously exposed to nicotinamide and etoposide (38). miR-34a restoration alone confers drug resistance via the SIRT1-NFκB pathway in tumors with p53 deficiency, which renders the combination of an NF-κB inhibitor and miR-34a a promising therapeutic strategy (39). A previous study indicated that SIRT1 regulates the expression of several antioxidant genes in bovine aortic endothelial cells, including *MnSOD*, *Prx3*, *Prx5*, *Trx2*, *TR2*, and *UCP-2* (40), which may be involved in the p53-independent pathway.

In summary, our data has demonstrated, for the first time to the best of our knowledge, that miR-34a reduces the migratory and invasive ability of SW480 cells, and induces apoptosis and cell cycle arrest, in a synergetic manner with 5-FU. As previously reported, miR-34a plays an important role as an apoptotic mediator, by alleviating drug resistance of colorectal cancer cells through the SIRT1/p53 pathway. Our data clearly illustrates the therapeutic potential of miR-34a, especially in combination with 5-FU, in the treatment of colorectal cancer.
